# Identification and validation of a six-gene signature associated with glycolysis to predict the prognosis of patients with cervical cancer

**DOI:** 10.1186/s12885-020-07598-3

**Published:** 2020-11-23

**Authors:** Luya Cai, Chuan Hu, Shanshan Yu, Lixiao Liu, Xiaobo Yu, Jiahua Chen, Xuan Liu, Fan Lin, Cheng Zhang, Wenfeng Li, Xiaojian Yan

**Affiliations:** 1 Department of Obstetrics and Gynecology, The First Affiliated Hospital of Wenzhou Medical University, 2 Fuxue Road, Wenzhou, Zhejiang 325000 P.R. China; 2 Department of Orthopaedic Surgery, The Affiliated Hospital of Qingdao University, Qingdao, 266071 China; 3 Department of Chemoradiation Oncology, The First Affiliated Hospital of Wenzhou Medical University, Wenzhou, Zhejiang P.R. China; 4 Department of Dermatology, The First Affiliated Hospital of Wenzhou Medical University, Wenzhou, Zhejiang P.R. China

**Keywords:** Cervical cancer, Glycolysis, Prognosis, Gene signature

## Abstract

**Background:**

Cervical cancer (CC) is one of the most common gynaecological cancers. The gene signature is believed to be reliable for predicting cancer patient survival. However, there is no relevant study on the relationship between the glycolysis-related gene (GRG) signature and overall survival (OS) of patients with CC.

**Methods:**

We extracted the mRNA expression profiles of 306 tumour and 13 normal tissues from the University of California Santa Cruz (UCSC) Database. Then, we screened out differentially expressed glycolysis-related genes (DEGRGs) among these mRNAs. All patients were randomly divided into training cohort and validation cohort according to the ratio of 7: 3. Next, univariate and multivariate Cox regression analyses were carried out to select the GRG with predictive ability for the prognosis of the training cohort. Additionally, risk score model was constructed and validated it in the validation cohort.

**Results:**

Six mRNAs were obtained that were associated with patient survival. The filtered mRNAs were classified into the protective type (GOT1) and the risk type (HSPA5, ANGPTL4, PFKM, IER3 and PFKFB4). Additionally, by constructing the prognostic risk score model, we found that the OS of the high-risk group was notably poorer, which showed good predictive ability both in training cohort and validation cohort. And the six-gene signature is a prognostic indicator independent of clinicopathological features. Through the verification of PCR, the results showed that compared with the normal cervial tissuses, the expression level of six mRNAs were significantly higher in the CC tissue, which was consistent with our findings.

**Conclusions:**

We constructed a glycolysis-related six-gene signature to predict the prognosis of patients with CC using bioinformatics methods. We provide a thorough comprehension of the effect of glycolysis in patients with CC and provide new targets and ideas for individualized treatment.

**Supplementary Information:**

The online version contains supplementary material available at 10.1186/s12885-020-07598-3.

## Background

Cervical cancer (CC) is one of the most common gynaecological cancers, accounting for the fourth cause of cancer-related death in women [1]. According to global cancer statistics, in 2018, there were nearly 570 thousand new cases of CC worldwide, with approximately 310 thousand deaths [1]. In recent years, with the launch of the human papillomavirus (HPV) vaccination programme, the incidence of CC in developed countries has significantly decreased, but in developing countries, it is still on the rise [2], and the age of onset tends to be younger. In addition, a large proportion of patients with CC are found to be in an advanced stage, and at this time, treatment options are extremely limited, and side effects are more serious, with a 5-year survival rate of less than 20% [3–5]. Moreover, patients with the same clinical stage and pathological type tend to adopt the same treatment, but the prognosis of patients is different, which is mainly due to the genetic heterogeneity of patients. Therefore, it is necessary to identify effective biomarkers to predict the prognosis of patients with CC.

Aerobic glycolysis is a special mechanism of tumour cell metabolism, also known as the Warburg effect [6], which plays an important role in promoting the growth and metastasis of various tumours, including CC. Some studies have found that HPV protein can promote the development of cancer through the Warburg effect, and the Warburg effect may also contribute to the enhancement of virus replication ability in the early stage of HPV infection [7]. In addition, total lesion glycolysis (TLG) is a measure of tumour metabolic activity, and some retrospective studies have found that TLG was significantly related to the recurrence-free survival (RFS) and overall survival (OS) of patients with CC [8–10]. Moreover, some glycolytic enzymes have also been proven to be related to the prognosis of CC. For example, hexokinase-2 (HK2), an enzyme that catalyses the conversion of glucose into glucose-6-phosphate, is overexpressed in a variety of cancers, has a promoting effect on the occurrence and development of CC, and is significantly related to the prognosis of patients [11]. Lactate dehydrogenase A (LDHA) and phosphofructokinase P (PFKP) were found to be significantly correlated with progression-free survival (PFS) and OS in patients with CC, and the expression level of LDHA in recurrent tumours was significantly higher than that in nonrecurrent tumours [12]. Glyceraldehyde-3-phosphate dehydrogenase (GAPDH) is a classical glycolytic enzyme that has been reported to be significantly increased in CC [13]. However, single gene biomarkers cannot achieve a good prediction effect, and some studies have suggested that gene signatures may be a better choice for predicting patient outcomes.

By mining public databases, many relevant studies have found that the glycolysis-related gene (GRG) signature is closely associated with the prognosis of patients with cancer, such as lung adenocarcinoma [14], liver cancer [15], pancreatic ductal carcinoma [16] and endometrial carcinoma [17]. Nevertheless, there is no bioinformatics research on this in CC. Thus, in this study, we analysed the relationship between the GRG signature and CC through the University of California Santa Cruz (UCSC) Database, which helped us better assess the prognosis of patients and provided new insights for individualized treatment of patients with CC.

## Method

### Acquisition of mRNA expression dataset

**Fig. 1:**
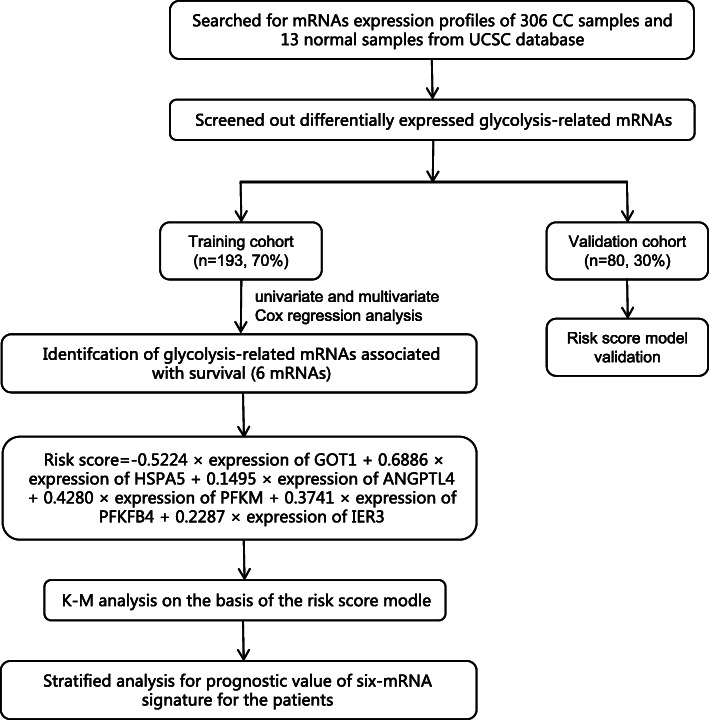
Flow chart of the bioinformatic analysis

The workflow of the present study is displayed as Fig. 1. We extracted the mRNA expression profiles of 306 CC samples and 13 normal samples from the UCSC database (http://xena.ucsc.edu/) (Supplementary Table 1). For UCSC dataset, RNA-seq data (FPKM values) were normalized into log2 (FPKM+ 1). Then, patients with OS less than 30 days were excluded, and 273 CC patients were included. The clinical information of CC patients were collected which included age, grade, American Joint Committee on Cancer (AJCC) stage, T classification, N classification, M classification, and OS (Supplementary Table 2).

### Identification and analysis of differentially expressed glycolysis-related genes (DEGRGs)

To obtain the cancer-related genes, we compared the expression levels of 16,208 mRNAs between CC and normal tissues using the limma package. A gene with false discovery rate (FDR) < 0.05 and |logFC| > 1 was defined as the DEG. Then, a list of GRGs (GO_GLYCOLYTIC_PROCESS, HALLMARK_GLYCOLYSIS, KEGG_GLYCOLYSIS_GLUCONEOGENESIS, REACTOME_GLYCOLYSIS, and BIOCARTA_GLYCOLYSIS_PATHWAY) was downloaded from the Molecular Signatures Database v5.1 (MSigDB). The Venn diagram was to identify the DEGRGs by combining GRGs and DEGs. Furthermore, to understand the potential function and related pathway of DEGRGs, Gene Ontology (GO) and Kyoto Encyclopedia of Genes and Genomes (KEGG) pathway enrichment analyses were carried out by the “clusterprofiler” package.

### Construction and validation of the prognostic risk score signature

Previous researches indicated that GRGs have potential prognostic value for cancer patients, but the role in CC patients remains unclear. Therefore, the survival analyses of GRGs in CC patients were performed in the present study. First, according to the ratio of 7: 3, all CC patients were randomly divided into training cohort (193 patients) and validation cohort (80 patients) (Table 1). In our research, the prognostic signature was developed in the training cohort and validated in the validation cohort. To identify the prognostic value of DEGRGs in CC patients, we performed univariate Cox regression analysis to confirm OS-related DEGRGs in training cohort. Then, based on the OS-related DEGRGs, a stepwise model selection by the Akaike information criterion (AIC) was used to avoid overfitting, and significant genes were incorporated into the multivariate Cox analysis to construct the GRG-based prognostic signature. By linear combination of the expression values of the selected genes weighted with the regression coefficients of the multivariate Cox regression analysis, the prognostic risk score model was established as follows:

$$ Risk\ \mathrm{Score}=\sum \limits_{i=0}^n{\beta}_i\ast {\mathrm{G}}_i $$

(*β*_*i*_ is the coefficient of the gene; *i* in multivariate Cox analysis; G_*i*_ represents the expression value of gene *i*; *n* is the number of genes in the signature).

**Table 1:** Clinicopathological parameters of patients with CC in the training cohort and validation cohort

Clinical characteristic	Total (***N*** = 273) N (%)	Training cohort (***N*** = 193) N (%)	Validation cohort (***N*** = 80) N (%)
Age			
< 50	171 (62.6)	124 (64.2)	47 (58.8)
> 50	102 (37.4)	69 (35.8)	33 (41.2)
Grade			
G1-G2	142 (52)	103 (53.4)	39 (48.8)
G3-G4	105 (38.5)	72 (37.3)	33 (41.2)
NA	26 (9.5)	18 (9.3)	8 (10)
T classification			
T1-T2	192 (70.3)	139 (72)	53 (66.3)
T3-T4	26 (9.5)	17 (8.8)	9 (11.2)
NA	55 (20.1)	37 (19.2)	18 (22.5)
N classification			
N0	118 (43.2)	92 (47.7)	26 (32.5)
N1	53 (19.4)	32 (16.5)	21 (26.3)
NA	102 (37.4)	69 (35.8)	33 (41.2)
M classification			
M0	101 (37)	69 (35.8)	32 (40)
M1	10 (3.7)	8 (4.1)	2 (2.5)
NA	162 (59.3)	116 (60.1)	46 (57.5)
AJCC stage			
I-II	209 (76.6)	150 (77.7)	59 (73.8)
III-IV	58 (21.2)	38 (19.7)	20 (25)
NA	6 (2.2)	5 (2.6)	1 (1.2)

*Abbreviations*: *T* Tumor, *N* Node (regional lymph node), *M* Metastasis

The time-dependent receiver operating characteristic (ROC) curve and area under the curve (AUC) value were used to evaluate the discrimination of the prognostic model. Additionally, the optimal cutoff value of risk score was determined by performing X-tile software, and patients were divided into low- and high-risk groups. Kaplan-Meier (K-M) survival curve and the log-rank test were adopted to compare the prognosis between two groups.

To further study the prognostic value of GRG signature in an independent cohort, the risk score of each patient in the validation cohort. Time-dependent ROC curve, AUC value, and K-M survival curve were used to show the prognostic ability of GRG signature in the validation cohort.

### Development of a clinical-GRG nomogram for CC patients

As reported in previous studies, several clinical variables were confirmed as prognostic variables, such as AJCC, T classification, N classification and M classification. Therefore, to develop a comprehensive prognostic model for CC patients, we firstly studied the prognostic value of clinical data, including age, grade, ATCC stage and TNM classification in CC patients by univariate Cox analysis. Moreover, the OS-related clinical data and GRG signature were incorporated into the multivariate Cox analysis and the independent variables were used to develop a nomogram. Concordance-index(C-index) and calibration curve were used to evaluate the nomogram.

### Specimens sources

Ten cases of CC and 10 cases of normal cervical tissue were obtained from the Department of gynecology and obstetrics, the First Affiliated Hospital of Wenzhou Medical University. Informed consent was provided by all patients. This study was approved by the ethics committee of the First Affiliated Hospital of Wenzhou Medical University. All tissues were pathologically diagnosed and stored in − 80 °C for preservation.

### Conducting quantitative real time (qRT)-PCR on tissues

Total RNA was refined from clinical specimens by TRIzol® reagent (Vazyme, Nanjing, China), then, following the manufacturer’s protocol, a PrimeScript RT reagent kit (Vazyme) was used to reverse transcription. qRT-PCR was performed to evaluate the DEGRGs expression in different specimens using SYBR-Green Premix (Vazyme) with specifc PCR primers shown in Supplementary Table 3. With GAPDH as the internal control, and the Ct method (2-ΔΔCt) was used to normalize the relative genes expression values.

### Correlation analysis between immune cell infiltration and glycolysis

We adopted CIBERSORT [18], which is widely used to describe the immune cell composition of the gene expression profiles to explain the 22 immune cell subtypes. The global *P* value of each sample deconvolution was determined by CIBERSORT, and samples with CIBERSORT *P* < 0.05 was selected for further study. Thus, we included a total of 184 patients from 193 patients. To determine the relationship between immune cell infiltration and glycolysis, we divided 184 patients into high- and low-risk groups based on the prognostic risk score and compared the differences in immune cells between the two groups and displayed the results by heatmap and violin plot. Furthermore, we conducted univariate Cox regression, LASSO and multivariate Cox regression analyses to examine whether the risk score and immune cells can be used as independent factors to predict the prognosis of CC patients.

## Results

### Preliminary screening of glycolysis-related mRNA

**Fig. 2:**
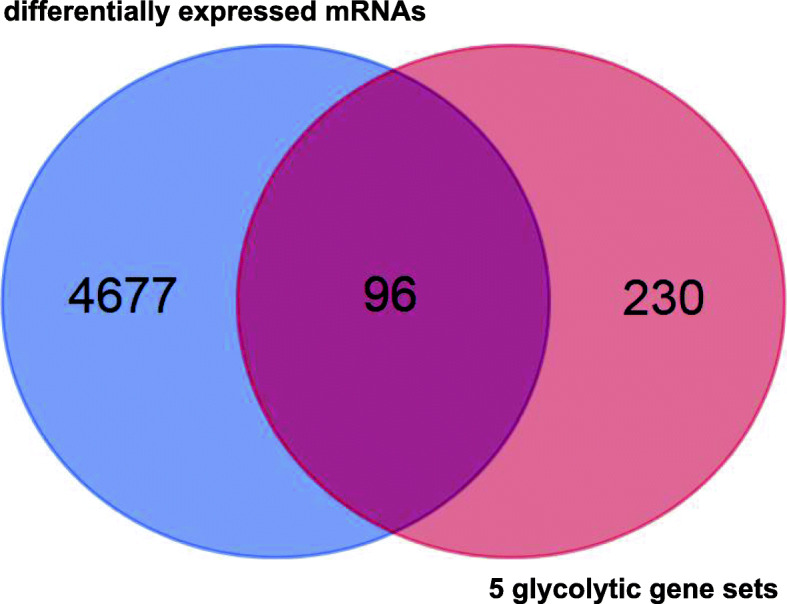
Identification of 96 DEGRGs in the differentially expressed mRNAs and 5 glycolytic gene sets (GO_GLYCOLYTIC_PROCESS, HALLMARK_GLYCOLYSIS, KEGG_GLYCOLYSIS_GLUCONEOGENESIS, REACTOME_GLYCOLYSIS, and BIOCARTA_GLYCOLYSIS_PATHWAY)

We obtained the mRNA expression profiles from the UCSC database, including 306 tumour samples and 13 normal samples. By comparing tumour and normal tissue samples, we screened 4772 differentially expressed mRNAs from 16,208 mRNAs. Then, Venn diagram software was applied to identify the DEGRGs in the 4772 differentially expressed mRNAs and 5 glycolytic gene sets, and the results showed that 96 DEGRGs were detected (Fig. 2).

**Fig. 3:**
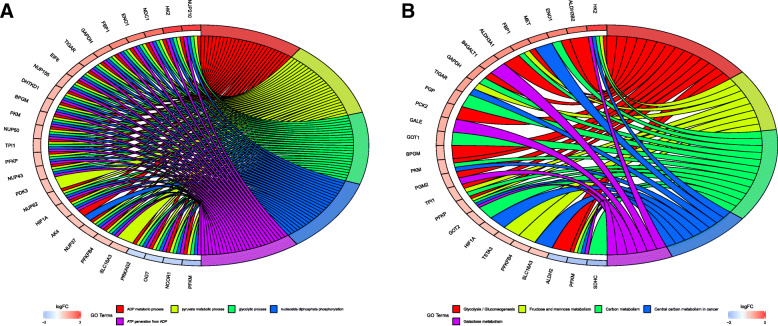
GO and KEGG pathway enrichment analysis of DEGRGs. **a** GO analysis. **b** KEGG pathway enrichment analysis. The right shows significantly enriched GO or KEGG terms, each bar on the left represents a gene and the depth of the color represents the logFC value of the gene. The intermediate line represents the connections between genes and GO or KEGG terms

To further analyse the relationship between these genes and glycolysis, we carried out GO analysis and KEGG pathway enrichment analysis on these genes. As a result, we found in GO analysis that the primary enriched terms were ADP metabolic process, pyruvate metabolic process and glycolytic process (Fig. 3a). For KEGG pathway enrichment analysis, glycolysis/gluconeogenesis, fructose and mannose metabolism and carbon metabolism were the most enriched. Based on these results, it has been shown that the genes we selected are indeed related to glycolysis (Fig. 3b).

### Identification of DEGRGs associated with survival and visualization of the DEGRG status

**Fig. 4:**
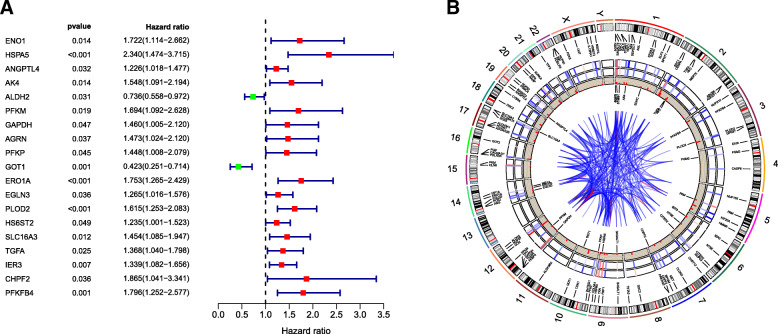
The results of univariate Cox analysis and circos analysis of DEGRG status in the whole genome. **a** Nineteen DEGRGs associated with survival. **b** The outermost layer shows 96 DEGRGs and their location in the whole genome, the middle part from the outside to the inside shows the expression of these genes in normal and tumour tissues, and the 19 DEGRGs associated with prognosis obtained by univariate Cox regression analysis in the training cohort. The inner layer is the PPI network. The medium confidence of the minimum required interaction score is 0.4, < 0.9 is blue, and > 0.9 is red

We analysed 96 mRNAs selected above to screen out survival-related mRNAs in training cohort by univariate Cox regression analysis and obtained 19 mRNAs with *P* <  0.05 (Fig. 4a). Next, we performed a stepwise model selection by the AIC, and six mRNAs (HSPA5, ANGPTL4, PFKM, GOT1, IER3 and PFKFB4) were obtained. Finally, multivariate Cox analysis was carried out to further identify the relationship of selected mRNAs with CC patients’ prognosis and obtained the coefficients. The filtered mRNAs were classified into the protective type (GOT1), whose HR < 1 was related to better prognosis, and the risk type (HSPA5, ANGPTL4, PFKM, IER3 and PFKFB4), whose HR > 1 was related to poor prognosis (Table 2).

**Table 2:** Information on six prognostic mRNAs significantly related to overall survival in patients with CC

mRNA	Β (Cox)	HR	95%CI of HR	P
HSPA5	0.6886	1.99	1.14–3.48	0.015
ANGPTL4	0.1495	1.16	0.96–1.41	0.125
PFKM	0.428	1.53	0.95–2.48	0.081
GOT1	−0.5224	0.59	0.34–1.05	0.072
IER3	0.2287	1.26	0.99–1.60	0.064
PFKFB4	0.3741	1.45	0.97–2.18	0.070

In addition, to examine the DEGRG status of the whole genome, we visualized the data by using Circos plots [19], which are shown in Fig. 4b. The outer layer includes chromosomes and 96 DEGRGs. The middle four layers, from the outside to the inside, are the average expression values of DEGRGs in normal tissues, the average expression values of DEGRGs in tumour tissues, the logFC of the difference analysis, and the 19 meaningful prognostic DEGRGs obtained by univariate Cox regression analysis in the training cohort. The inner layer is the protein-protein interaction (PPI) network of the DEGRGs. In addition, 0.4 was defined as the minimum required interaction score, and between 0.4 and 0.9 is displayed in blue, while greater than 0.9 is displayed in red.

### Constructing and validating a six-mRNA signature to predict patient prognosis

**Fig. 5:**
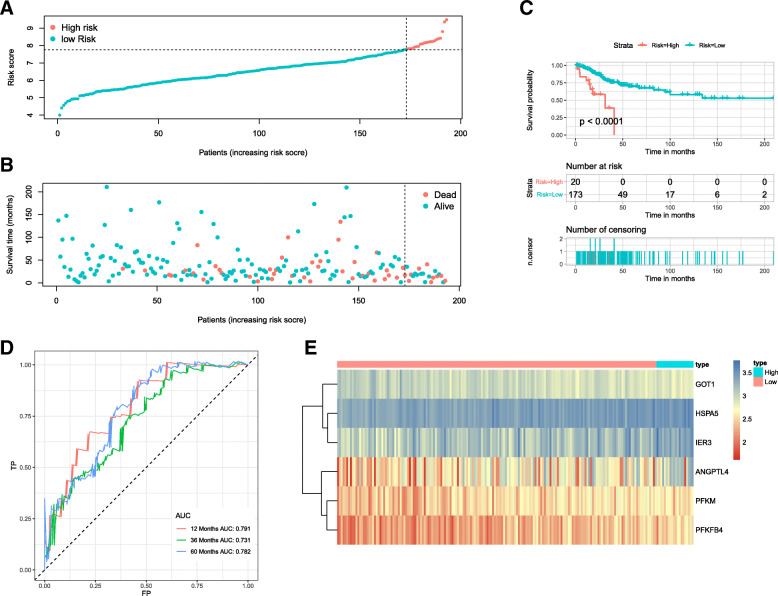
The six-mRNA signature associated with risk score predicts OS in the training cohort. **a** mRNA risk score distribution. **b** Survival days of patients. **c** K-M curve to test the predictive effect of the gene signature in the training cohort. **d** ROC curve analysis to evaluate the sensitivity and specificity of the gene signature. **e** A heatmap of the expression profile of six mRNAs

Using the linear combination of the expression value of the selected genes and the regression coefficient of multivariate Cox regression analysis, the following predictive risk scoring model was established: risk score = − 0.5224 × expression of GOT1 + 0.6886 × expression of HSPA5 + 0.1495 × expression of ANGPTL4 + 0.4280 × expression of PFKM + 0.3741 × expression of PFKFB4 + 0.2287 × expression of IER3. Based on the prognosis risk score, 193 patients were divided into high- and low-risk groups by using optimal risk score cutoff identified by X-tile as the boundary value (Fig. 5a), and the respective survival status of 193 patients was presented (Fig. 5b). The K-M analysis showed that compared to the high-risk group, the OS in the low-risk group was dramatically better (*p* < 0.0001; Fig. 5c). The AUCs for 1-, 3- and 5-year OS were 0.791, 0.731 and 0.782, respectively (Fig. 5d), suggesting that the six-mRNA signature has excellent diagnostic significance for prognosis prediction. Additionally, we generated a heatmap to exhibit the expression profiles of the six mRNAs, from which we can see that, compared with the low-risk group, the expression level of risk-type mRNAs (HSPA5, ANGPTL4, PFKM, IER3 and PFKFB4) of the high-risk group was apparently higher, while the expression level of the protective-type mRNA GOT1 was opposite (Fig. 5e).

**Fig. 6:**
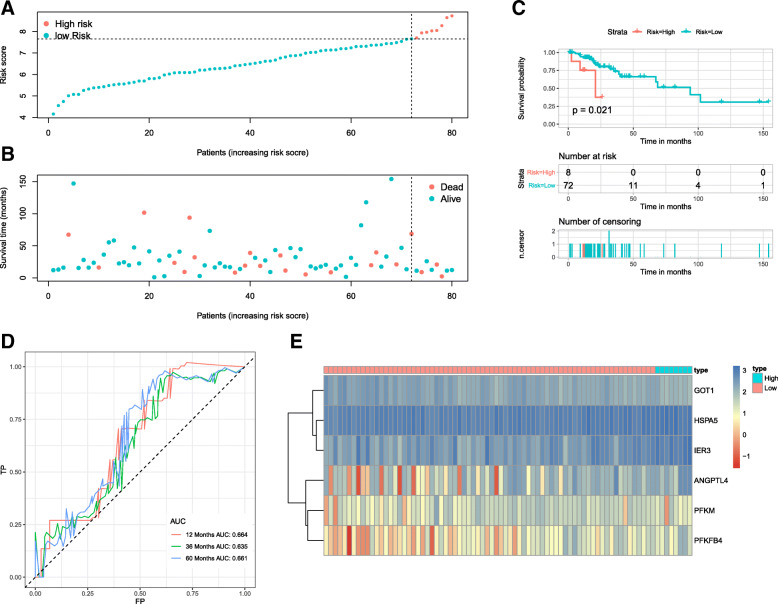
Validation of the six-mRNA signature in the validation cohort. **a** mRNA risk score distribution. **b** Survival days of patients. **c** K-M curve to test the predictive effect of the gene signature in the validation cohort. **d** ROC curves of the gene signature. **e** A heatmap of the expression profile of six mRNAs

Next, we validated the predictive ability of the six-mRNA signature in the validation cohort. Eighty patients were divided into high- and low-risk groups by using the same optimal risk score cutoff which were used in the training cohort (Fig. 6a), and the respective survival status of 80 patients was presented (Fig. 6b). And the K-M analysis showed that the patients in the low-risk group had significantly better OS (*P* = 0.021) (Fig. 6c), which AUC values were 0.664, 0.635 and 0.661 for 1-, 3- and 5-year, respectively (Fig. 6d).

### The risk score constructed by the six-mRNA signature is an independent prognostic indicator

**Fig. 7:**
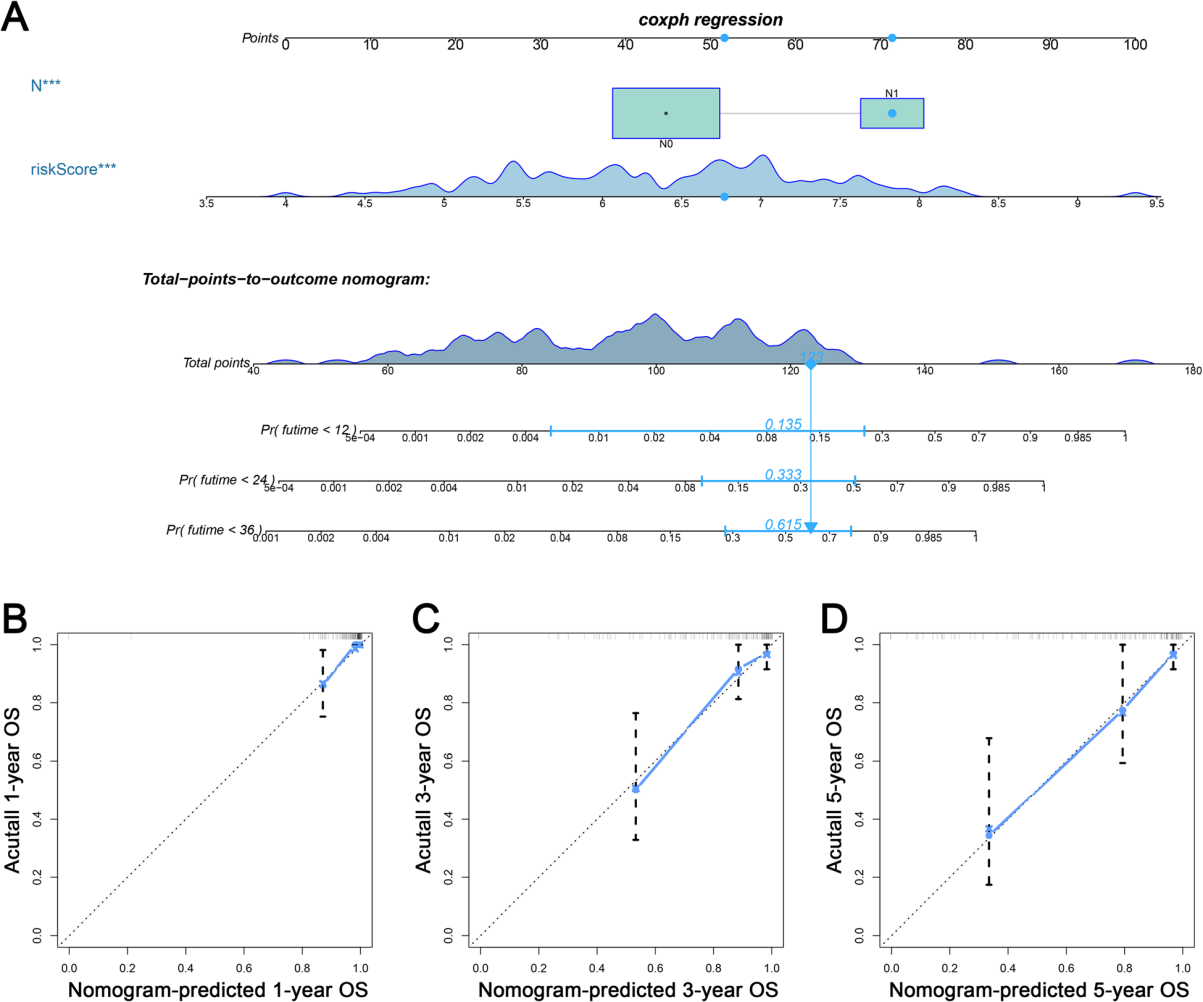
The establishment of a nomogram which can predict the prognosis probability of CC patients. **a** OS nomogram was constructed combined with risk score and N classification. **b**-**d** The calibration plot of OS nomogram for 1-, 3- and 5-year survival

To evaluate whether the six-mRNA signature was an independent prognostic indicator, we performed univariate and multivariate Cox regression analyses to compare the prognostic values of risk score with other clinical characteristics. We included a total of 193 patients, and among these patients, 124 (64.2%) were younger than 50 years old, 69 (35.8%) were older than 50 years old. Among 193 patients, 103 (53.4%) had grade 1–2 tumours, and 72 (37.3%) had grade 3–4 tumours, 139 (72.0%) had T1-T2 tumours, 17 (8.8%) had T3-T4 tumours. Furthermore, among these patients, 92 (47.6%) had no lymph node metastasis, 32 (16.5%) had lymph node metastasis, 69 (35.7%) had no distant metastasis, and 8 (4.1%) had distant metastasis. Additionally, among these patients, 150 (77.7%) had stage I-II disease, and 38 (19.7%) had stage III-IV disease. In all the above data, except for age and grade, AJCC stage (HR = 2.78, 95% CI 1.55–4.98, *p* < 0.001), T classification (HR = 5.15, 95% CI 2.55–10.42, *p* < 0.001), M classification (HR = 4.70, 95% CI 1.53–14.50, *p* = 0.007) and N classification (HR = 4.18, 95% CI 1.83–9.53 *p* < 0.001) showed significant differences in the univariate Cox regression analysis, which can be used as independent prognostic factors (Table 3). In the next multivariate Cox regression analysis, only the N classification showed significant differences (HR = 14.80, 95% CI 3.44–63.76, *p* < 0.001) (Table 3). However, regardless of univariate or multivariate Cox regression analysis, the risk score showed significant prognostic value (HR = 2.72, 95% CI 1.92–3.85, *p* < 0.001; HR = 2.57, 95% CI 1.47–4.49, *p* = 0.002) (Table 3). Based on these results above, we constructed a nomogram prediction model combined the risk score with N classification to predict CC patients’ OS (Fig. 7a), which C index is 0.83. The calibration plot showed that in the nomogram, the predicted values of OS at 1, 3 and 5 years for CC patients have a good correlation with the actual values (Fig. 7b-d).

**Table 3:** Univariable and multivariable Cox regression analysis for each clinical feature

Clinical feature	Univariate analysis			Multivariate analysis		
	HR	95%CI of HR	***P*** value	HR	95%CI of HR	***P*** value
Risk score	2.72	1.92–3.85	< 0.001	2.57	1.47–4.49	0.002
Age (≤50/> 50)	1.37	0.78–2.41	0.276	–	–	–
Grade (G1-G2/G3-G4)	1.03	0.55–1.93	0.925	–	–	–
T (T1-T2/T3-T4)	5.15	2.55–10.42	< 0.001	–	–	–
M (M0/M1)	4.70	1.53–14.50	0.007	–	–	–
N (N0/N1)	4.18	1.83–9.53	< 0.001	14.80	3.44–63.76	< 0.001
AJCC stage (I-II/III-IV)	2.78	1.55–4.98	< 0.001	–	–	–

*Abbreviations*: *T* Tumor, *N* Node (regional lymph node), *M* Metastasis, *HR* Hazard ratio, *95% CI* 95%Confidence Interval

**Fig. 8:**
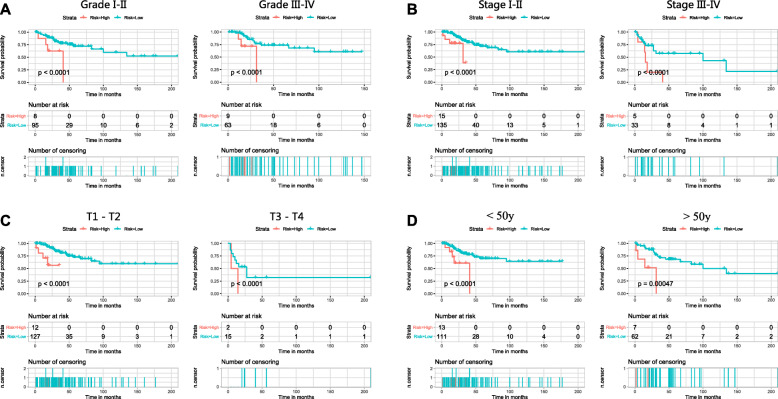
Stratified analysis for the prognostic value of the six-mRNA signature for patients. **a** Grade, **b** AJCC stage, **c** T classification, **d** Age

Next, stratified analysis was performed to further validate whether the six-mRNA signature can be used as an independent prognostic factor, according to the grade, AJCC, T classification and age. First, we classified patients into the grade 1–2 dataset and grade 3–4 dataset, and the patients in the datasets were divided into high- and low-risk groups, respectively. As a result, we found that there were significant differences in OS between these two groups, and the OS of the low-risk group was obviously better (Fig. 8a). Next, we did the same thing for the other clinicopathological features, and we found that the OS of the high-risk group was significantly worse than that of the low-risk group regardless of the AJCC stage (stage I-II or stage III-IV) (Fig. 8b), T classification (T0 or T1) (Fig. 8c) and age (younger than 50 or older than 50) (Fig. 8d), further confirming the reliability of our analysis.

### Validating the overexpression of the six mRNAs in CC tissues by qRT-PCR

**Fig. 9:**
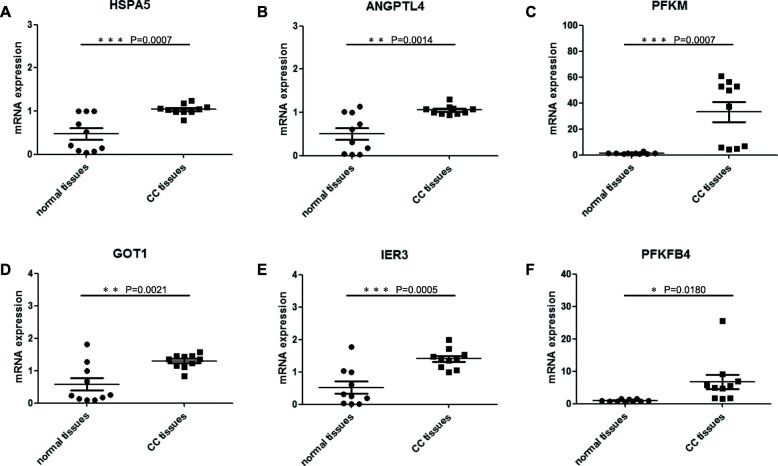
Expressions of six mRNAs in CC tissues and normal tissues. **a** HSPA5, **b** ANGPTL4, **c** PFKM, **d** GOT1, **e** IER3, **f** PFKFB4

We validated the expression level of six mRNAs in 10 CC tissues and 10 normal cervical tissues by using qRT-PCR. The results showed that compared with the normal cervial tissuses, the expression level of HSPA5, ANGPTL4, PFKM, GOT1, IER3 and PFKFB4 were significantly higher in the CC tissue (Fig. 9), making the bioinformatics analysis result much more reliable and precise.

### The relationship between immune cell infiltration and glycolysis

**Fig. 10:**
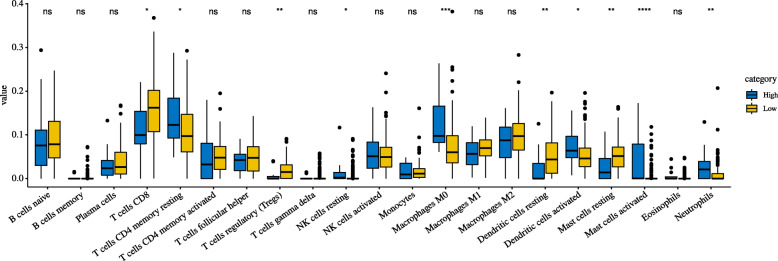
Box plots for comparison of immune cell infiltration between high-risk and low-risk groups

We analysed a total of 184 patients’ immune cell composition from their gene expression profiles. Additionally, we divided these patients into two groups, and we can see that the expression levels of CD8 T cells, regulatory T cells (Tregs) and resting mast cells were significantly higher in the low-risk group compared to the high-risk group; however, in the high-risk group, the expression levels of neutrophils, M0 macrophages, activated mast cells and resting CD4 memory T cells were dramatically higher (Fig. 10).

In addition, we analyzed the relationship between immune cells and prognosis of CC patients. In the univariate Cox regression analysis, CD8 T cells, resting CD4 memory T cells, M0 macrophages, M2 macrophages, resting mast cells, activated mast cells and Neutrophils all showed significant differences with *p* < 0.05. Among these immune cells, the HR of CD8 T cells, M2 macrophages and resting mast cells were less than 1, which associated with better prognosis. In the next multivariate Cox regression analysis, only activated mast cells showed significant differences with *p* < 0.05. And both in univariate or multivariate Cox regression analysis, the risk score showed significant prognostic value (*p* < 0.001) (Table 4).

**Table 4:** Univariable and multivariable Cox regression analysis for immune cells

Immune cells	Univariate analysis			Multivariate analysis		
	HR	95%CI of HR	***P*** value	HR	95%CI of HR	***P*** value
Risk Score	2.58	1.80–3.71	< 0.001	2.11	1.43–3.11	< 0.001
T cells CD8	0.002	3.89E-05 - 0.10	0.002	–	–	–
T cells CD4 memory resting	174.15	3.19–9503.14	0.011	–	–	–
Macrophages M0	3081.50	23.73–400,288.68	0.001	–	–	–
Macrophages M2	0.00	9.46E-08 - 0.22	0.018	–	–	–
Mast cells resting	0.00	1.14E-10 - 0.05	0.011	–	–	–
Mast cells activated	2.43E-06	207,417.10–2.4E+ 11	< 0.00122925.08	6.23 - 8E+ 07	0.017	
Neutrophils	2.23E+ 8	81.22–5.28E+ 10	0.005	–	–	–

## Discussion

In recent years, increasing attention has been paid to the relationship between energy metabolism and tumours, among which the Warburg effect is a notable feature of the energy metabolism of tumour cells. Despite the low efficiency of glycolysis, tumour cells are still active in glycolysis, which can produce more energy and various metabolites in a short time so that tumour cells can benefit from glycolysis [6]. More importantly, the rapid growth of tumours is often accompanied by hypoxia, and hypoxia inducible factor (HIF), as the key regulator of glycolysis, can solve the nutritional needs of tumour cells by inducing the expression of glucose and amino acid transporters (glucose transporter type 1 (GLUT1)) and L-type amino acid transporter 1 (LAT1) to further promote the progression of cancer [20, 21]. There have been many studies on the relationship between glycolysis and tumours, but the relationship between GRGs and the prognosis of patients with CC is still very limited. Because a single gene biomarker cannot provide a strong prediction effect, a more accurate and reliable gene signature was used to predict the clinical outcomes of patients. For instance, a glycolysis-related nine-gene signature was used to predict the survival of patients with endometrial cancer [17]. A tumour microenvironment-related nine-gene signature was used to predict overall survival with ovarian cancer [22]. Moreover, an autophagy-related five-gene signature was developed for prognosis prediction in patients with prostate cancer [23]. In this study, we used the GRG signature for the first time to predict the survival of patients with CC and obtained a good prediction effect.

First, we screened out the differentially expressed glycolytic mRNAs from the CC dataset in the UCSC database and selected six DEGRGs with predictive ability for the prognosis of patients with CC in the training cohort through univariate and multivariate Cox regression analysis (*P* < 0.05). Subsequently, by constructing the prognostic risk score model, we found that there was a significant difference in OS between the high- and low-risk groups, and the OS of the high-risk group was evidently worse. Furthermore, the risk score model was validated in the validation cohort with good predictive ability. Finally, further verification was done through PCR, we found that the expression of these six genes in tumor tissue was indeed significantly higher than that in normal tissue.

From our analysis, we selected six DEGRGs (HSPA5, ANGPTL4, PFKM, GOT1, IER3 and PFKFB4). Heat shock protein A5 (HSPA5, also konwn as GRP78), a member of the HSP70 family, has been found to play an important role in cancer malignancy and anti-tumor therapy. And it has been reported that the high expression of HSPA5 can protect cancer cells from immune surveillance, and inhibition of its expression can promote cell apoptosis and inhibit tumor growth [24, 25]. Angiopoietin-like4 (ANGPTL4) is highly expressed in various tumors, which is closely related to tumor growth and metastasis, such as hepatocellular carcinoma [26], breast cancer [27], head and neck squamous cell carcinoma [28] and melanoma [29]. And ANGPTL4 participates in the construction of GRG signature in lung adenocarcinoma to predict the survival and metastasis of patients [14]. In addition, previous studies have found that ANGPTL4 is significantly associated with the susceptibility of CC, is a potential risk factor [30]. Muscular phosphofructokinase (PFKM), a member of the phosphofructokinase (PFK) family, can promote the growth of muscle-infiltrating bladder cancer [31] and can be used as a new breast cancer gene [32], and its expression level can distinguish normal tissues from CC tissues [33]. 6-Phosphofructo-2-kinase/fructose-2,6-biphosphatase 4 (PFKFB4), is a key kinase in Warburg pathway [34], has been found to be associated with a variety of cancers, including breast cancer [35], prostate cancer [36] and glioblastoma [37], promoting the progression and metastasis of cancer, and may become an effective molecular target of anti-tumor drugs. It was found that the expression of immediate early response gene 3 (IER3) was increased in advanced cancer [38, 39], but some studies have found that IER3 can also promote tumour cell apoptosis and has anti-tumour activity, such as lower expression in CC tissues [40], and increasing the expression of IER3 can enhance the sensitivity of CC cells to radiotherapy [41]. In contrast, in our study, we found that IER3 exists as a risk factor. Moreover, glutamate oxaloacetate transaminase 1 (GOT1), is a gene that encodes cytoplasmic aspartate aminotransferase and a key aspartate-producing protein. Glutamine metabolism is essential for the proliferation of cancer cells in addition to glucose. Glutamine derived glutamic acid is used to generate nonessential amino acids by GOT1 in high proliferative cells, which plays an important role in cell proliferation [42, 43]. Moreover, HIFɑ can inhibit the proliferation of tumor cells by inhibiting the synthesis of aspartate [44]; however, in our study, we found that GOT1 was a protective factor, with higher expression in the low-risk group than in the high-risk group. Hence, further studies are needed to explore the relationship between these two genes (IER3 and GOT1) and the prognosis of patients with CC.

Interestingly, we also found some divergences in immune cell infiltration between the high- and low-risk groups, suggesting that glycolysis may be correlated with tumour immunity. In addition, several previous studies have elucidated the relationship between glycolysis and immunity. Excess glycolysis will lead to acidic tumor microenvironment, affect the infiltration of immune cells in varying degrees, and contribute to the survival of cancer cells [45]. Cascone et al. found that in patients with melanoma and NSCLC, high glycolytic activity will be accompanied by poor immune cell infiltration, such as cytoxic T cells, T helper cells, memory T cells, macrophases, or natural killer (NK) cells reduction [46]. Li et al. found that high glycolysis group had a higher immunescore and higher TIL percentage in breast cancer, however, the immune cells with anti-tumor effect were not enriched, and concluded that high glycolysis was associated with immunosuppression of tumor microenvironment [47]. In our study, the expression levels of CD8 T cells and resting mast cells were significantly higher in the low-risk group. CD8 T cells, to our knowledge, play a pivotal role in the control of tumour cell growth, and their relationship with the prognosis of patients with CC has also been confirmed in a previous study [48, 49]. For mast cells, some studies have found that mast cell infiltration in CC indicates poor clinical prognosis [49–52], which was consistent with our findings that resting mast cells were highly expressed in the low-risk group and that activated mast cells were highly expressed in the high-risk group. These findings are consistent with previous studies and to some extent explain why patients in the low-risk group have better survival outcomes.

It is undeniable that this study does have some defects. On the one hand, we obtained clinical gene information for only 273 cases of CC, and the sample size was not large enough. On the other hand, it would be better to have an external validation cohort to verify the accuracy of the prediction model rather than the internal validation cohort. However, we verified these six DEGRGs between tumor and normal tissues, which to some extent compensated for this defect.

In summary, we first identified the relationship between the GRG signature and the prognosis of patients with CC using bioinformatics methods and found that patients in the high-risk group had significantly lower OS than those in the low-risk group. Furthermore, we found some relationships between the infiltration of immune cells and glycolysis.

## Conclusion

In brief, we constructed a six-gene signature (HSPA5, ANGPTL4, PFKM, GOT1, IER3 and PFKFB4) to predict the prognosis of patients with CC, which was also a prognostic factor independent of clinicopathological features. These findings will provide us with new insights into the role of glycolysis in CC, guiding individualized treatment for patients with CC.

## Supplementary Information

**Additional file 1: Table S1.** The mRNA expression profiles.

**Additional file 2: Table S2.** The clinical information of 273 patients with CC from the UCSC database.

**Additional file 3: Table S3.** The specifc PCR primers of six mRNAs.

## Data Availability

The datasets generated and/or analysed during the current study are available in the the University of California Santa Cruz (UCSC) Database and Molecular Signatures Database, [http://xena.ucsc.edu/, https://www.gsea-msigdb.org/gsea/msigdb/].

## Abbreviations

CC: Cervical cancer
HPV: Human papillomavirus
TLG: Total lesion glycolysis
RFS: Recurrence-free survival
OS: Overall survival
PFS: Progression-free survival
GAPDH: Glyceraldehyde-3-phosphate dehydrogenase
GRG: Glycolysis-related gene
UCSC: University of California Santa Cruz
DEGRGs: Differentially expressed glycolysis-related genes
GO: Gene Ontology
KEGG: Kyoto Encyclopedia of Genes and Genomes
AJCC: American Joint Committee on Cancer
ROC: Receiver operating characteristic
HIF: Hypoxia inducible factor
GLUT1: Glucose transporter type 1
PFKM: Phosphofructokinase
EGFRs: Epidermal growth factor receptors
IER3: Immediate early response gene 3
GOT1: Glutamate oxaloacetate transaminase 1
PDAC: Pancreatic ductal adenocarcinoma
